# The Association between Hypoxia-Induced Low Activity and Apoptosis Strongly Resembles That between TTX-Induced Silencing and Apoptosis

**DOI:** 10.3390/ijms23052754

**Published:** 2022-03-02

**Authors:** Domitilla Taxis di Bordonia e Valnigra, Gerco C. Hassink, Marloes R. Levers, Monica Frega, Jeannette Hofmeijer, Michel J. A. M. van Putten, Joost le Feber

**Affiliations:** 1 Clinical Neurophysiology, Faculty of Science and Technology, University of Twente, 7500 AE Enschede, The Netherlands; domitillataxis@gmail.com (D.T.d.B.e.V.); g.c.hassink@utwente.nl (G.C.H.); m.r.levers@utwente.nl (M.R.L.); m.frega@utwente.nl (M.F.); j.hofmeijer@utwente.nl (J.H.); m.j.a.m.vanputten@utwente.nl (M.J.A.M.v.P.); 2 Department of Neurology, Rijnstate Hospital, 6800 TA Arnhem, The Netherlands; 3 Department of Clinical Neurophysiology, Medisch Spectrum Twente, 7500 KA Enschede, The Netherlands

**Keywords:** in vitro model, ischemic penumbra, electrophysiological activity, apoptosis, live staining, TTX

## Abstract

In the penumbra of a brain infarct, neurons initially remain structurally intact, but perfusion is insufficient to maintain neuronal activity at physiological levels. Improving neuronal recovery in the penumbra has large potential to advance recovery of stroke patients, but penumbral pathology is incompletely understood, and treatments are scarce. We hypothesize that low activity in the penumbra is associated with apoptosis and thus contributes to irreversible neuronal damage. We explored the putative relationship between low neuronal activity and apoptosis in cultured neurons exposed to variable durations of hypoxia or TTX. We combined electrophysiology and live apoptosis staining in 42 cultures, and compared effects of hypoxia and TTX silencing in terms of network activity and apoptosis. Hypoxia rapidly reduced network activity, but cultures showed limited apoptosis during the first 12 h. After 24 h, widespread apoptosis had occurred. This was associated with full activity recovery observed upon reoxygenation within 12 h, but not after 24 h. Similarly, TTX exposure strongly reduced activity, with full recovery upon washout within 12 h, but not after 24 h. Mean temporal evolution of apoptosis in TTX-treated cultures was the same as in hypoxic cultures. These results suggest that prolonged low activity may be a common factor in the pathways towards apoptosis.

## 1. Introduction

Ischemic stroke is the second most common cause of death worldwide and the main cause of long-term neurological disability in adults [1]. Within the ischemic territory, the region with perfusion less than 8–10 mL/100 mg/min is referred to as the core. Here, blood supply is insufficient to maintain ion gradients across the neuronal plasma membrane and loss of neuronal function is quickly followed by neuronal death. In surrounding areas, often referred to as the ‘penumbra’, perfusion levels are higher, between 10 and 35 mL/100 g/min, because of blood supply from adjacent arteries [2]. Here, neurons initially remain structurally intact and viable, but activity is impeded by widespread failure of synaptic transmission. Synaptic failure in the penumbra is one of the early consequences of mild-to-moderate cerebral ischemia [3,4]. The penumbra is viable for at least several hours, depending on remaining perfusion levels, and may be salvaged by timely restoration of perfusion, resulting in improved patient outcomes [2]. If reperfusion is not established in time, the neurons in the penumbra undergo transition to irreversible neuronal damage [5,6,7].

Characterization of mechanisms driving the transition from reversible to irreversible neuronal damage in the penumbra is of primary importance for identification of new treatment targets. However, the pathophysiological mechanisms that underlie the transition towards recovery or neuronal death in the penumbra are incompletely understood. Apoptosis is a common final path towards cell death in the penumbra, which may occur several hours or days after stroke onset [8,9,10,11]. Thus, prevention of apoptotic cell death may reduce neuronal loss [12].

Recent studies in in vitro models showed that a wide range of hypoxic depths induce disturbances of synaptic neurotransmission, with a consequent suppression of neuronal network activity, without neuronal depolarization [13,14]. Under these conditions of mild-to-moderate hypoxia, time to transition from reversible synaptic failure to irreversible neuronal damage appeared rather constant, uncorrelated with hypoxic depth [14]. Time courses of activity reduction also appeared relatively independent of hypoxic depths, suggesting low neuronal activity as a possible factor that may contribute to progression to cell death in critically perfused brain areas, such as the penumbra [15,16].

The most common type of cell death in the ischemic penumbra is apoptosis [8,17]. Apoptotic signaling is generally classified as proceeding by either an intrinsic or an extrinsic pathway. Intrinsic apoptosis is initiated by microenvironmental perturbations such as growth factor withdrawal, DNA damage, reactive oxygen species overload, or mitotic defects. Extrinsic apoptosis can be driven by death receptors, whose activation depends on the binding of the associated ligand(s), or dependence receptors, whose activation occurs when the levels of their specific ligand drop below a specific threshold [18].

If low activity contributes to apoptosis in the ischemic penumbra, this should be reflected when activity is suppressed pharmacologically. Several studies showed that low neuronal activity induced by tetrodotoxin (TTX) is also associated with apoptosis [19,20], but other work did not find such association [21,22], possibly due to differences in experimental preparation. Pharmacological disinhibition, leading to increased network activity, was shown to prevent apoptosis in developing cortical neurons [23]. The hypothesis of activity-dependent neuronal survival is further supported by in vitro studies showing that neuronal activation restored synaptic density after hypoxia [24], and that electrical or optogenetic stimulation during hypoxia improved neuronal survival [25]. 

The association between low activity and apoptosis may involve insufficient activation of voltage-sensitive calcium channels [19,26] and insufficient expression of brain-derived neurotrophic factor (BDNF). BDNF release is activity-dependent [27] and is mediated by voltage-controlled calcium channels [26]. Low BDNF may lead to apoptosis through intrinsic as well as extrinsic pathways. Normal levels of BDNF suppress intrinsic apoptosis by activation of TrkB receptors, which triggers downstream signaling mechanisms that inhibit the pro-apoptotic proteins Bax and Bad [28]. On the other hand, p75 neurotrophin receptors, to which BDNF can bind, have been shown to function as dependence receptors in extrinsic apoptosis [29].

In the current study, we further investigated the putative relationship between low neuronal activity and apoptosis in an in vitro model of the ischemic penumbra [13,14,16,25]. If low activity in this model indeed contributes to the transition to apoptosis, pharmacological suppression of activity in this preparation should reflect that. Electrophysiological activity was recorded in parallel with live staining for apoptosis in cultures of dissociated cortical neurons that were exposed to hypoxia or to TTX, a potent selective sodium channel blocker. We studied the association between low activity and apoptosis during hypoxia and TTX. In addition, we determined the ‘point of no return’: the maximum duration of hypoxia- or TTX-induced low activity until full recovery was no longer observed. 

## 2. Results

### 2.1. Activity and Apoptosis during Hypoxia

**Figure 1:**
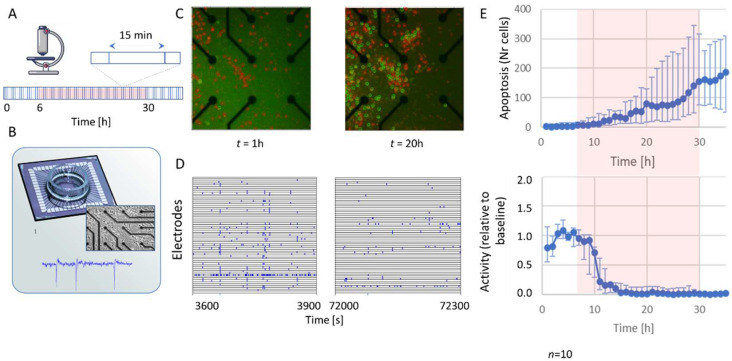
Simultaneous assessment of electrophysiological activity and apoptosis in neuronal cultures exposed to hypoxia. (**A**) Experimental protocol: fluorescent images were taken from rat cortical cultures stained for apoptosis and cell death every 15 min during baseline (normoxia; 6 h), hypoxia (10% of normoxia; 24 h, indicated by red-shaded background), and after reoxygenation (normoxia; 6 h). (**B**) Micro-electrode Array (MEA) system for recording of electrophysiological activity. Insets show a phase-contrast image of cells on the MEA and an example of a recorded signal. (**C**) Example photographs of a culture stained for apoptosis (green) and cell death (red), taken at 1 h (left; baseline), or 20 h (right; hypoxia). Apoptotic and dead cells were automatically detected and counted by a custom computer algorithm (green and red circles). (**D**) Examples of 5 min of recorded activity after 1 h (left; normoxia) or 20 h (right; hypoxia). Vertical axes show traces of the 60 electrodes, all blue ticks represent action potentials. Hypoxia greatly reduced the amount of action potentials and synchronicity. (**E**) Median temporal evolution of apoptosis (upper) and activity (lower panel) in 10 cultures. Error bars represent 25% and 75% percentiles.

Simultaneous measurement of neuronal activity and apoptosis was performed in ten cultures (23.4 ± 1.1 DIV) before, during, and after exposure to 24 h of hypoxia. Figure 1 shows an example of recorded activity (Figure 1D) and apoptosis (Figure 1C) in one of the cultures at times *t* = 1 h (before hypoxia) and *t* = 20 h (during hypoxia). Figure 1E shows the median temporal evolution of activity and apoptosis for ten cultures. The number of apoptotic cells remained very low during the first hours of hypoxia, and then started to increase. The time of onset and the rate at which apoptosis developed differed between cultures, as reflected by the relatively large error bars at *t* > 20 h (Figure 1E, upper panel). Activity and number of active electrodes quickly dropped during hypoxia and showed no recovery upon reoxygenation after 24 h. Burstiness index (*BI*) during baseline averaged 0.5 ± 0.2. The total number of action potentials recorded during and after hypoxia was not sufficient to calculate *BI* in these phases.

### 2.2. Activity and Apoptosis during TTX

Thirty-two experiments (23.9 ± 1.1 DIV) were analyzed to evaluate the relation between inactivity duration and recovery, subdivided into experiments of 3 h (*n* = 5), 6 h (*n* = 6), 12 h (*n* = 4), or 24 h (*n* = 4) of TTX exposure. Upon addition of TTX to the bath, *AWFR* dropped to less than 1% of baseline *AWFR* in all cultures. For 3 h, 6 h, and 12 h, *n* = 3 control cultures underwent the same treatment, except the addition of TTX. We used *n* = 4 control cultures for 24 h experiments.

#### 2.2.1. Three and Six Hours of TTX

**Figure 2:**
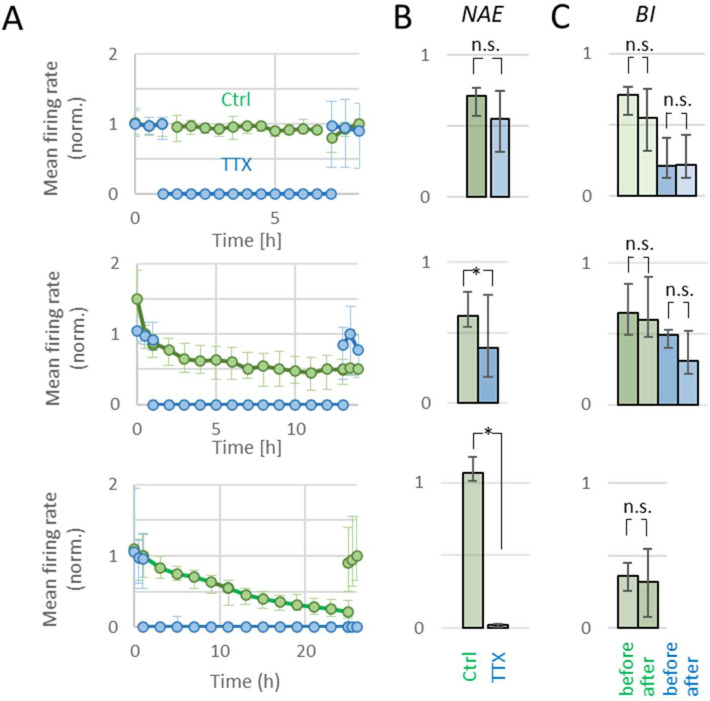
Effect of TTX exposure on neuronal activity. (**A**) Temporal evolution of mean firing rate before, during, and after exposure to TTX (blue) and under control conditions (green), for 6 h (top panel), 12 h (middle), or 24 h (bottom). (**B**) Number of active electrodes (*NAE*) after washout in TTX (blue) and control recordings (green), normalized to *NAE* during baseline. (**C**) Burstiness index (*BI*) before (left) and after (right) exposure to TTX (blue) or control conditions (green). After 24 h of TTX, there was no recovery of activity and *BI* could not be determined. * indicates significant difference (*p* < 0.05); n.s.: not significant.

TTX exposure for three or six hours gave very similar results, with complete loss of activity during TTX and full recovery of *AWFR* and *BI* after washout. Figure 2A (top) shows the temporal evolution of *AWFR* during and after 6 h exposure to TTX. In four cultures that were exposed to TTX for 6 h, activity recorded 24 h after washout was used for analysis (see Methods). After washout, *AWFR* showed complete recovery to pre-TTX values (*p* > 0.42). *BI* could not be calculated during TTX blockade, but values after washout did not differ from baseline (*p* > 0.87). *NAE* dropped to 0 during TTX and recovered after washout. No apoptosis was observed in 3 h or 6 h TTX experiments.

In all 3 h control experiments, activity remained present immediately after ‘washout’. In one 6 h control experiment, activity recorded 24 h after ‘washout’ was used for analysis. In all 3 h and 6 h control recordings, *AWFR*, *BI*, and *NAE* were restored to baseline values after washout. No apoptosis was observed in 3 h or 6 h control experiments.

#### 2.2.2. Twelve Hours of TTX

Figure 2A (middle) shows the temporal evolution of *AWFR* during and after 12 h exposure to TTX. Twelve hours of TTX treatment completely blocked all activity in all cultures, as observed by *AWFR* = 0 and *NAE* = 0. In one TTX-treated culture, activity remained very low immediately after washout and the activity recorded 24 h after washout was used for analysis. *AWFR* and *NAE* recovered after washout, although *NAE* remained at ~30% below baseline *NAE* (*p* < 0.01). *BI* after washout was unaffected by 12 h of TTX, compared to baseline (*p* > 0.32). Twelve hours of exposure to TTX slightly enhanced apoptosis (*p* < 0.05). This increase was not seen in control cultures. In contrast to shorter recordings, *AWFR* and *NAE* slightly decreased (*p* < 0.04) during 12 h control recordings, followed by complete recovery after washout (*p* > 0.82 and *p* > 0.48, respectively). *BI* remained unchanged during 12 h control recordings. 

**Figure 3:**
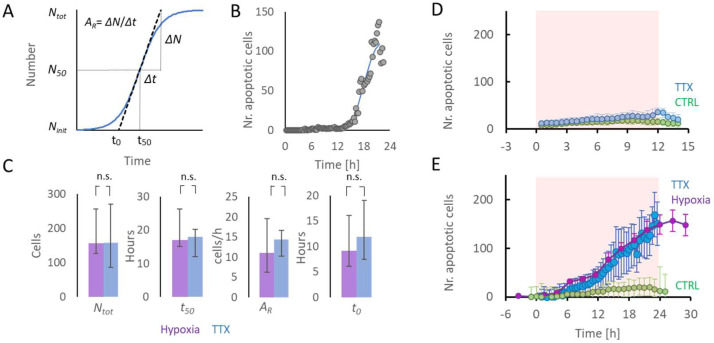
Temporal evolution of the number of apoptotic cells in cultures exposed to hypoxia (purple), TTX (blue), or control conditions (green). (**A**) A sigmoid function (Equation (1)) was fitted to the temporal evolution of the number of apoptotic cells to estimate the apoptotic rate (*A_R_*), the maximum number of apoptotic cells (*N_tot_*), and the time until 50% of *N_tot_* has been reached (*t_50_*). These parameters were used to estimate the time of onset of apoptosis, *t_0_* (Equation (2)). (**B**) Example of temporal evolution of number of apoptotic cells in a culture exposed to TTX for 24 h. (**C**) Median values of *N_tot_*, *t_50_*, and *A_R_* for cultures exposed to hypoxia (*n* = 13, purple) or TTX (blue, *n* = 9) for 24 h. n.s.: not significant. Error bars represent 25–75% quartiles. (**D**) Averaged temporal evolution of apoptosis in cultures under control conditions (green; *n* = 4) or exposed to TTX (blue; *n* = 3) for 12 h. (**E**) Averaged temporal evolution of apoptosis in cultures under control conditions (green; *n* = 4), or exposed to TTX (blue; *n* = 11) or hypoxia (purple; *n* = 17) for 24 h. Error bars indicate SEM. Shaded orange background in (**D**) and (**E**) indicates exposure to TTX or hypoxia.

Figure 3D shows the temporal evolution of apoptosis in cultures exposed to 12 h of TTX and control cultures. Apoptosis in TTX-treated cultures remained low, but was higher than in control cultures (*p* < 0.05).

#### 2.2.3. Twenty-Four Hours of TTX

Figure 2A (bottom) shows the temporal evolution of *AWFR* during and after 24 h exposure to TTX. *AWFR* significantly decreased to ~30% of baseline during 24 h control recordings (*n* = 4), but remained higher than *AWFR* in TTX-treated cultures (*n* = 4). After washout, *AWFR* recovered to baseline values in control recordings (*p* > 0.50), but not in TTX-treated cultures (*p* < 0.02, see Figure 2). *NAE* and *BI* remained unaffected in 24 h control experiments, while *NAE* significantly dropped to almost zero in TTX-treated cultures, and showed no recovery after washout (*p* < 0.01).

No significant apoptosis occurred during 26 h in control experiments, while the number of apoptotic cells increased significantly during 24 h of TTX treatment (*p* < 0.02). 

### 2.3. Initiation of Apoptosis Associated with TTX- or Hypoxia-Induced Low Activity 

The association between duration of low activity induced by TTX (*n* = 11) or hypoxia (*n* = 17) and apoptosis was analyzed in 28 cultures. On average, temporal evolutions of apoptosis in TTX and hypoxia experiments were very similar (Figure 3E). A sigmoid function (Equation (1)) was fitted to the temporal evolution of the number of apoptotic cells to determine *N_tot_*, *N_50_*, *A_R_*, and the time of apoptosis onset, *t_0_* (Equation (2)). Two TTX experiments and four hypoxia experiments were excluded from this analysis because the fit error was too large (55 ± 36% of *N_tot_*). The average fit error of all included experiments (*n* = 9 + 13) was 11.6 ± 6.4%. Exposure to TTX or hypoxia yielded the same median values for *N_tot_* (157 vs. 159; *p* > 0.99) and *T_50_* (17 h vs. 18 h; *p* > 0.85). The apoptotic rate (A*_R_*) tended to be slightly higher after TTX exposure, but differences were not significant (*p* > 0.3). Times of onset of apoptosis (*t_0_*) were not significantly different (9.1 vs. 11.8 h *p* > 0.4, see Figure 3C).

### 2.4. Relation between Remaining Activity during Hypoxia and Apoptosis

**Figure 4:**
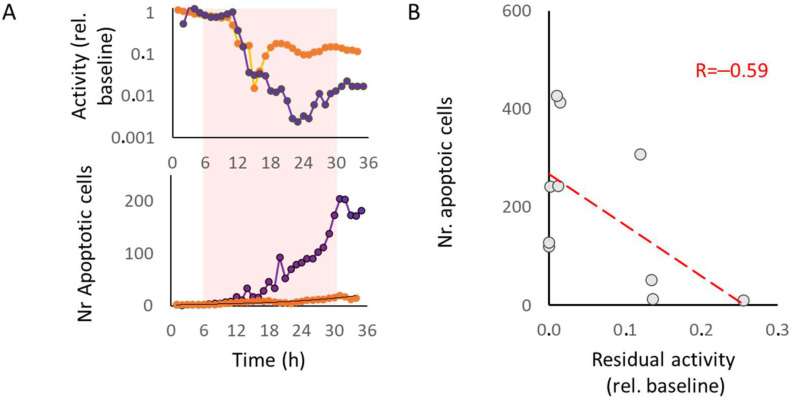
Temporal evolution of apoptosis and residual activity in cultures exposed to 24 h of hypoxia. Activity decreased in all cultures during hypoxia, but residual activity varied between cultures. (**A**) Examples of cultures with relatively high (orange) and relatively low (purple) residual activity (top panel) during hypoxia (10% of normoxia, light-red area). Fewer cells became apoptotic (bottom panel) in the culture with more residual activity than in the culture with less activity. (**B**) When all cultures were grouped, the average amount of remaining activity during the second half of the hypoxic phase (*t* = 18–30 h) negatively correlated with the number of apoptotic cells at *t* = 35 h. Red dashed line indicates fitted linear trend line (R = −0.59, *p* = 0.07).

In ten of the cultures exposed to hypoxia, apoptosis and activity were recorded in parallel. In all cultures, activity decreased during hypoxia, but the remaining level of activity varied between cultures. Figure 4A shows two examples with different residual activity during hypoxia. The average remaining activity during the last 12 h of hypoxia (as a fraction of baseline activity) negatively correlated with the number of apoptotic cells as counted after the hypoxic period at *t* = 35 h (R = −0.59, *p* = 0.07, see Figure 4B).

## 3. Discussion

In this study, we used cultures of dissociated neurons to investigate apoptosis in relation to neuronal activity. We lowered neuronal activity temporarily by hypoxia or TTX and observed more apoptosis with longer low activity. Neuronal activity recovered after up to 12 h of TTX-induced inactivity, but not after 24 h. Comparably, in cultures exposed to hypoxia, activity did not recover upon reoxygenation after 24 h. Under either condition, apoptosis started after 9–12 h. These results suggest a relationship between low neuronal activity and apoptosis, but it is difficult to infer causality. In cultures exposed to hypoxia, low activity may have contributed to apoptosis, or vice versa. In TTX-treated cultures, low activity preceded apoptosis, but we cannot exclude that TTX induced apoptosis via another, independent, molecular pathway. Nevertheless, the strong similarity of the temporal evolution of apoptosis between both conditions shows a close connection between low activity and apoptosis. We hypothesize that reduced neuronal activity, as may occur in the ischemic penumbra of stroke patients, may contribute to apoptosis. 

Several studies suggest that activity dependent apoptosis is an important process to shape the developing cerebral cortex. Large regional differences are seen in the early post-natal density of apoptotic neurons, which correlate negatively to regional activity [30]. Suppression of activity by ketamine was shown to enhance apoptosis during early brain development, which could be reversed by elevating neuronal activity [31]. Blockade of NMDA receptors during late fetal or early neonatal life triggered widespread apoptotic neurodegeneration in the developing rat brain [32]. Ethanol, acting by blockade of NMDA receptors and excessive activation of GABA_A_ receptors, triggered widespread apoptotic neurodegeneration in the developing rat forebrain [33,34]. It was concluded that any drug that modifies physiological activity patterns during early development may have an immediate impact on apoptosis [35].

The duration of TTX-induced neuronal silence that is associated with apoptosis in in vitro neuronal networks is debated and may differ between brain areas. Heck et al. found a significant increase in the number of apoptotic neurons in organotypic cortical slices as early as 6 h after TTX–induced silencing [19]. Segal and colleagues also found apoptotic cell death in very young TTX-silenced cultures of dissociated cortical neurons, but on longer timescales [36,37]. In contrast, multiple days of TTX silencing in hippocampal cultures did not result in apoptosis [21,22].

Interestingly, addition of AMPA antagonists protected TTX-silenced cortical neurons from degenerating. Electrophysiological recordings showed very large miniature excitatory postsynaptic currents (mEPSCs) that were blocked by AMPA receptor antagonists, suggesting that upscaled mEPSCs may contribute to apoptosis in otherwise chronically silenced neurons [36]. The potential of glutamatergic antagonists to prevent apoptosis associated with inactivity was supported by a recent study that showed that three weeks of neuronal silencing by TTX, combined with glutamatergic antagonists, did not lead to apoptosis in cortical cultures [38].

Whereas TTX exposure of healthy cortical networks is associated with apoptosis, TTX was shown to reduce apoptosis in cortical cultures exposed to hypoxia [39]. A similar result was found in gerbils after bilateral occlusion of the common carotid arteries [40]. These observations may seem contradictory to the results presented here, but in those studies the hypoxic/ischemic burden was substantially more severe and induced neuronal depolarization, as reflected by the observed high intracellular Na^+^ [39]. Under these conditions, the potency of TTX to contribute to restoration of the membrane potential probably dominated its effect on network activity.

In the current study, apoptosis started after ~12 h of TTX-induced neuronal silence. Hypoxia, at the depth applied here, initially did not affect membrane integrity, but it strongly reduced activity due to large-scale synaptic failure [13,14]. In contrast to TTX, hypoxia did not completely block all activity. Still, apoptosis tended to occur slightly earlier in cultures exposed to hypoxia than in TTX-treated cultures, suggesting that there are additional factors that may enhance apoptosis under hypoxic conditions. Despite the earlier onset, the apoptotic rate tended to be lower in hypoxic cultures, resulting in a number of apoptotic cells after 24 h that was very similar to that following TTX-silencing. Apoptosis under both conditions clearly exceeded that under control conditions (normoxia, no TTX). Temporal evolution of apoptosis under control conditions was recorded here in four cultures. This was deemed sufficient because the observed negligible apoptosis confirms numerous observations that most cultures remain healthy under control conditions much longer than the 3–4 weeks of incubation used here [41,42,43,44]. The higher apoptotic rate after TTX silencing may be associated with higher ATP availability. Apoptosis requires ATP [45,46], which is scarce under hypoxic conditions but widely available during TTX treatment. 

Lower levels of network activity usually included less synchronized network bursts. Recent work suggests that not only the level of residual activity may play a role in the onset of apoptosis, but also the occurrence of synchronized network bursts [47]. This is in agreement with an earlier study that showed that pharmacologically induced reduction in spontaneous burst activity in organotypic slices and dissociated cultures led to an increase in caspase 3-dependent apoptosis [48]. Whether and how reduced synchronicity of firing patterns is essential for the association between low activity and apoptosis remains an open question.

Twenty-four hours of neuronal silencing by TTX showed no activity recovery upon washout. Normoxic control recordings of the same duration showed slowly decreasing activity, possibly related to slow depletion of glucose [47]. Here, activity remained higher than in TTX-treated cultures and medium change after 24 h resulted in full recovery of activity, suggesting the level of residual activity as a determinant for activity recovery. This is in agreement with the finding that higher residual activity during hypoxia was associated with less apoptosis (Figure 4). Recent work demonstrated the association between hypoxia duration and activity recovery [14]. After up to 12 h of hypoxia, activity returned to values comparable to baseline and also connectivity was restored [16], but not after 24 h or longer. The current study revealed the same relationship between the duration of low activity and activity recovery in TTX-silenced cultures, suggesting that the duration of insufficient activity is another determinant for activity recovery.

The association between hypoxia and apoptosis has also been observed in other disorders, including epilepsy, particularly during prolonged seizures [49]. The mechanism of action here remains unclear, but may involve excitotoxicity, with hyperactivity leading to hypoxia [50]. Thus, whereas both disorders show an association between hypoxia and apoptosis, the mechanism of action during prolonged seizures probably differs from that in the ischemic penumbra, where hypoxia/ischemia causes and precedes low activity. 

After activity blockade of 3 h, 6 h, or 12 h, the amount of activity fully recovered following washout. However, 12 h of TTX-induced silencing reduced the number of active electrodes. Hence, remaining electrodes recorded more activity than during baseline. This may reflect reduced inhibition: reduced inhibitory activity would enhance firing of other neurons at the active electrodes, while electrodes that recorded from lost inhibitory neurons might become inactive. This view is in agreement with earlier studies that demonstrated higher vulnerability of inhibitory neurons to hypoxia [14,49], and a decreasing inhibitory synapse density [51,52].

Several differences between in vitro models and the in vivo brain may hamper translation of the current findings to the brain, including a significant difference in pO_2_ levels. In this study, neurons were cultured under atmospheric pO_2_ of almost 150 mmHg, while in vivo the pO_2_ ≈ 35 mmHg [53,54]. Nevertheless, cultures showed an immediate response to decreased pO_2_, showing a clear effect of pO_2_ variations. Another difference between our model and cerebral ischemia is the relatively high availability of glucose in our model. The presence of glucose allows the anaerobic production of ATP, which is limited if both oxygen and glucose are depleted. This was considered acceptable because we aimed to strongly reduce the available ATP, but not to zero. Network composition (i.e., ratio between excitatory and inhibitory neurons and neuron-to-astrocyte ratio) was not explicitly controlled in the current study, and may have affected results. However, the culturing technique used yields networks that, on average, resemble network composition in the in vivo cortex [16]. Furthermore, we used random mixtures of male and female cells, which have been shown to express minor differences between apoptotic pathways at early developmental stages and different vulnerability to moderate (but not severe) hypoxia in mature mice [55]. Differing fractions of male and female cells may have contributed to the variation between experiments.

In conclusion, low activity induced by TTX or hypoxia is associated with apoptosis at essentially similar timescales, with onsets after 9–12 h. Activity fully recovered upon washout or reoxygenation within 12 h, but not after longer exposure to TTX or hypoxia. The similar associations between low activity and apoptosis or recovery are in agreement with the hypothesis that prolonged low neuronal activity may play a role in the transition to cell death. This finding does not prove a causal relationship between low activity and apoptosis, but it is a precondition for verification of the hypothesis, and suggests that low activity may be a common factor in the pathways towards apoptosis.

## 4. Materials and Methods

### 4.1. Cell Cultures

Newborn Wistar rats (male and female) were decapitated within 35 h after birth, and brain cortices were collected to obtain cells for culturing. After trypsin treatment, the cortices were dissociated by trituration and cultured in R12H medium. Around 100,000 dissociated neurons were plated on a multi-electrode array (MEA; Multi Channel System, Reutlingen, Germany) precoated with polyethylene imine (PEI). The electrode area of the MEA was surrounded by a glass ring (Ø20 mm) to create a culture chamber that could contain 1000 µL of R12H medium [56]. Cultures growing on MEAs developed in an incubator under standard conditions of 36 °C, high humidity, and 5% CO_2_. Twice a week, half of the medium was refreshed. Experiments were performed after 3 weeks of culturing, when networks had matured [57,58] and cell density reached confluence. All experiments were conducted according to Dutch law and approved by the Dutch committee on animal use (CCD; Project number AVD110002016802).

### 4.2. Maintenance of Normoxia and Induction of Hypoxia

During measurements, MEAs were covered with an O_2_ and CO_2_ permeable membrane to avoid evaporation and to protect them from contamination [44]. MEAs were placed in the recording setup (MEA2100, Multi Channel Systems, 72770 Reutlingen, Germany) under a Plexiglas hood (V ≈ 0.25 L) and a humidified gas mixture of air and N_2_ completed with 5% CO_2_ was blown over the setup at a rate of 0.4 L/min. This flowrate was sufficient to maintain CO_2_ at 5% as confirmed by the constant color of the pH indicator in the growth medium. Mixtures of air and N_2_ could be set at any ratio and were computer-controlled by mass flow controllers (Vögtlin Instruments, 4147 Aesch BL, Switzerland). Normoxic conditions were realized by 100% air and no N_2_. The hypoxic mixture contained 10% air and 90% N_2_. In the first 15 min after a change in composition of the mixture, the flow rate was 5 L/min to shorten the transition time. Switching to the hypoxic mixture was shown to reduce the partial oxygen pressure in the culture medium from ~160 mmHg (normoxia) to ~20 mmHg (hypoxia) [14]. This restricted the available amount of adenosinetriphosphate (ATP), thus modeling the limited perfusion in the ischemic penumbra. In the penumbra of a brain infarct, the availability of glucose and oxygen are both compromised. Adjustment of glucose availability would require a change of medium. In our experimental protocol, we chose to restrict the available amount of oxygen but not glucose. This facilitated smooth adjustments of ATP availability without interrupting recordings or interfering with culture sterility. Temperature was maintained constant for the entire experiment at 36 °C.

### 4.3. Data Acquisition

Cells were cultured on Micro-Electrode Arrays (Multi Channel Systems 72770 Reutlingen, Germany) with 60 embedded microelectrodes arranged in an 8 × 8 matrix, excluding the four electrodes at the corners (TiN, 30 µm diameter, 200 µm spacing). Recordings were obtained at a sample rate of 25 kHz per channel using MC Rack (Multi Channel Systems, 72770 Reutlingen, Germany). All analog signals were band-pass filtered with cut off frequencies of 200 and 3300 Hz. Spikes were detected whenever the signal crossed a threshold, initially set at 5.5 times the standard deviation of noise recorded at that electrode. Thresholds were inspected visually to ensure that only action potentials were detected that clearly exceeded noise fluctuations, and adjusted if necessary. Detection thresholds were set before the start of the experiment and were not updated during recording. Timestamps and channel numbers of each detected event were stored, as well as 6 ms wave shapes (2 ms before–4 ms after the detection). 

#### 4.3.1. Artifact Detection

To remove possible artifacts that were collected together with cells’ activity, an offline analysis based on an algorithm adapted from Wagenaar was performed [59]. In short, a detected event was accepted as a recorded action potential if it was the largest deflection from 0 (of either polarity) within a 1 ms window around the detected event, and there were no secondary peaks of the same polarity and more than 50% of the amplitude of the putative action potential within that same window. To avoid false detections due to possible drift in noise amplitude during experiments and the stationary detection threshold in the MEA2100 setup, the data acquired with this system were subjected to a second threshold. Of all detected events, 6 ms of waveshape were stored and the root mean square of the signal given by the first 1.5 ms and the last 2 ms was computed. To be considered valid, it was necessary that the amplitude of the event was at least 5.5 times this value, otherwise the detection was discarded.

#### 4.3.2. Exclusion Criteria

Only active and bursting cultures were used for experiments. A culture was considered active if, on average, at least 500 spikes/min were recorded during baseline. All cultures that were included showed at least 2 network bursts per minute during baseline, resulting in mean baseline *BI* ≥ 0.1 (see below).

### 4.4. Readouts

#### 4.4.1. Array-Wide Fire Rate (*AWFR*)

The array-wide fire rate (*AWFR*) is the summed number of action potentials of all electrodes in 1 h time bins. It was used to determine the amount of activity and to evaluate activity recovery in each culture. To enable comparison between experiments, the *AWFR* was normalized to the mean baseline value for each culture. 

#### 4.4.2. Burstiness Index (*BI*)

The burstiness index (*BI*) is useful to give information about synchronicity of neurons. It was defined as the fraction of the total number of spikes accounted for by the 15% of bins with the largest counts, *f*_15_, in 5 min recording divided into 1 s-long time bins. This value was normalized from 0 (no bursts) to 1 (burst dominated) as *BI* = (*f*_15_–0.15)/0.85 [59]. It was not computed for activity recorded during TTX exposure when mean *AWFR* was lower than 5% of baseline *AWFR*.

#### 4.4.3. Number of Active Electrodes (*NAE*)

The number of active electrodes (*NAE*) was defined as the number of electrodes that recorded at least 60 spikes in 1 h time bin. *NAE* was normalized to the baseline value for each culture to enable comparison between experiments. 

#### 4.4.4. Apoptosis Imaging

PI (ThermoFisher Scientific, Waltham MA, USA) was used to stain dead cells. A stock solution was made at a concentration of 0.25 mM, and 2 µL of this stock was added to the (1 mL) culture bath to achieve a final concentration of 0.5 µM. caspase 3/7 staining (ThermoFisher Scientific, Waltham MA, USA) was used to visualize apoptotic cells. A 1 µL measure of the 2 mM stock solution was added to the culture bath, yielding a final concentration of 2 µM. This fluorogenic substrate for activated caspase 3 and 7 gives a green fluorescent response with a maximum absorption of 502 nm. PI and caspase 3/7 were added to the cells before the start of baseline recordings, and again after washout procedures.

The TMD-EF fluorescence equipment mounted on the Nikon inverted microscope was used with a 10× magnification objective to evaluate the expression of PI and caspase 3/7 staining. Fluorophores were excited using a mercury lamp. Automated photography at fixed time intervals of 15 min was controlled by a custom LabView program. To avoid bleaching, preparations were only exposed to the light when pictures were taken. The pictures were acquired on a computer in remote mode, using the publicly available program DigiCamControl. Exposure time was 0.4 s. 

A MATLAB script was used to automatically count the stained apoptotic cells in all acquired images. In short, images of 3264 × 3264 pixels were 2D moving average-filtered (for each pixel, the average was taken of squares of 41 × 41 pixels symmetrically surrounding the pixel). If local maxima in the filtered image exceeded a threshold, set at 70% of the mean green intensity, it was counted as an apoptotic cell.

### 4.5. Pharmacologically Induced Inactivity

Neuronal network inactivity was induced pharmacologically by adding tetrodotoxin (TTX, 1 µM, Abcam), a potent sodium channel blocker. All TTX experiments were conducted under normoxic conditions. Experiments consisted of one hour of baseline, followed by 3 h, 6 h, 12 h, or 24 h of inactivity induced by TTX. 

Then, TTX was washed out: all medium was removed from the MEA, and the culture was washed three times with fresh medium (R12H). Then, 1 mL of conditioned medium, with 1 µL of caspase and 1 µL of PI, was added to the MEA. Conditioned medium consisted of 500 µL of medium collected from the same culture during the previous medium refreshment and 500 µL of fresh R12H medium. Conditioned medium was stored at 4 °C and warmed up to 36 °C shortly before being added to the culture. 

After washout, the MEA was repositioned such that the photographed region was the same as during baseline and TTX phase. MEA electrodes were used for navigation. Again, 1 h of spontaneous activity was recorded and the array-wide firing rate was calculated to quantify recovery of activity. In some cultures, activity recovery did occur, but not immediately after washout, possibly due to incomplete washout. Therefore, another hour of activity was recorded 24 h later to determine possibly delayed recovery. If activity recorded immediately after baseline was <5% of that recorded during baseline, the activity recorded 24 h after washout was used for further analysis.

Control experiments were performed for each of the durations (3, 6, 12, or 24 h). The same procedure was followed as in TTX experiments, except the actual addition of TTX to the bath. We will refer to these experiments as 3, 6, 12, or 24 h control recordings, although in fact recordings were longer in total, as they also included a baseline and ‘post washout’ recording.

### 4.6. Onset of Apoptosis 

To determine the duration of TTX-induced inactivity or hypoxia until the onset of apoptosis, a sigmoid function was fitted to the temporal evolution of the number of apoptotic cells, *N(t)*, using:

(1) $$N\left(t\right)=N_{init}+\frac{N_{tot}}{1+exp\left(\left(\frac{-4}{N_{tot}}\right)\cdot A_{R}\cdot \left(t-t_{50}\right)\right)}$$

Here, *N_init_* represents the initial number of apoptotic cells, *N_tot_* the estimated total number of cells that became apoptotic, and *t_50_* the time when *N_50_* (= (*N_tot_*–*N_init_*)/2) cells had become apoptotic. At this time, the number of apoptotic cells increased most rapidly. *A_R_* represents the rate at which apoptosis developed at time *t_50_*. The term $\frac{4}{N_{tot}}$ in the denominator facilitates interpretation of *A_R_* as the maximum steepness of the sigmoid, expressed in cells/h. For each experiment, *N_init_* was estimated by the average number of apoptotic cells in the first 5 images, and the parameters *N_tot_, t_50_,* and *A_R_* that minimized the mean squared error were computed using a Nelder–Mead simplex algorithm [60]. If the square root of the mean squared error exceeded 25% of *N_tot_*, the quality of the fit was considered insufficient and the experiment was excluded from this analysis. The estimated time of onset of apoptosis (*t_0_*, see Figure 3A) was calculated as:

(2) $$t_{0}=t_{50}-\frac{N_{50}}{A_{R}}$$

Finally, the medians and 25% and 75% quartiles of *t_50_*, *A_R_*, and *t_0_* were calculated and used to describe the temporal evolution of apoptosis. 

### 4.7. Statistical Analysis

Results in groups of cultures based on the treatment condition were represented by their mean and standard error of the mean (SEM) if their distribution passed a Shapiro–Wilk normality test. Otherwise, the median and 25 and 75 percentiles were used to visualize group results.

The comparisons between different treatment groups (TTX vs. control or TTX vs. hypoxia) were performed using a two-tailed Mann–Whitney test for readouts *AWFR*, *BI*, and *NAE*, while a Kruskal–Wallis test was used to evaluate the differences between these readouts in the phases baseline, TTX, and recovery or baseline, hypoxia, and re-oxygenation.

Pearson correlation coefficient (R) between Residual activity and number of apoptotic cells was determined as the ratio between the covariance of the variables and the product of their standard deviations.

All statistical analyses were performed in MATLAB (R2017b). *p* < 0.05 was considered to indicate significant differences. 

## Data Availability

The data presented in this study are available on request from the corresponding author.
